# The Spread of COVID-19 Crisis Communication by German Public Authorities and Experts on Twitter: Quantitative Content Analysis

**DOI:** 10.2196/31834

**Published:** 2021-12-22

**Authors:** Larissa S Drescher, Jutta Roosen, Katja Aue, Kerstin Dressel, Wiebke Schär, Anne Götz

**Affiliations:** 1 C³ team GbR Munich Germany; 2 TUM School of Management Technical University of Munich Freising Germany; 3 Süddeutsches Institut für empirische Sozialforschung e.V. Munich Germany

**Keywords:** COVID-19, crisis communication, content analysis, Twitter, experts, authorities, Germany, negative binomial regression, social media, communication, crisis, information, development

## Abstract

**Background:**

The COVID-19 pandemic led to the necessity of immediate crisis communication by public health authorities. In Germany, as in many other countries, people choose social media, including Twitter, to obtain real-time information and understanding of the pandemic and its consequences. Next to authorities, experts such as virologists and science communicators were very prominent at the beginning of German Twitter COVID-19 crisis communication.

**Objective:**

The aim of this study was to detect similarities and differences between public authorities and individual experts in COVID-19 crisis communication on Twitter during the first year of the pandemic.

**Methods:**

Descriptive analysis and quantitative content analysis were carried out on 8251 original tweets posted from January 1, 2020, to January 15, 2021. COVID-19–related tweets of 21 authorities and 18 experts were categorized into structural, content, and style components. Negative binomial regressions were performed to evaluate tweet spread measured by the retweet and like counts of COVID-19–related tweets.

**Results:**

Descriptive statistics revealed that authorities and experts increasingly tweeted about COVID-19 over the period under study. Two experts and one authority were responsible for 70.26% (544,418/774,865) of all retweets, thus representing COVID-19 influencers. Altogether, COVID-19 tweets by experts reached a 7-fold higher rate of retweeting (*t*_8,249_=26.94, *P*<.001) and 13.9 times the like rate (*t*_8,249_=31.27, *P*<.001) compared with those of authorities. Tweets by authorities were much more designed than those by experts, with more structural and content components; for example, 91.99% (4997/5432) of tweets by authorities used hashtags in contrast to only 19.01% (536/2819) of experts’ COVID-19 tweets. Multivariate analysis revealed that such structural elements reduce the spread of the tweets, and the incidence rate of retweets for authorities’ tweets using hashtags was approximately 0.64 that of tweets without hashtags (*Z*=–6.92, *P*<.001). For experts, the effect of hashtags on retweets was insignificant (*Z*=1.56, *P*=.12).

**Conclusions:**

Twitter data are a powerful information source and suitable for crisis communication in Germany. COVID-19 tweet activity mirrors the development of COVID-19 cases in Germany. Twitter users retweet and like communications regarding COVID-19 by experts more than those delivered by authorities. Tweets have higher coverage for both authorities and experts when they are plain and for authorities when they directly address people. For authorities, it appears that it was difficult to win recognition during COVID-19. For all stakeholders studied, the association between number of followers and number of retweets was highly significantly positive (authorities *Z*=28.74, *P*<.001; experts *Z*=25.99, *P*<.001). Updated standards might be required for successful crisis communication by authorities.

## Introduction

### Background

The occurrence of the SARS-CoV-2 virus and the ongoing COVID-19 pandemic have made crisis communication inevitable. Across countries, people use social media as their source of information about development of the pandemic [1]. Consequently, public authorities use different social media channels to deliver information about various aspects of the virus, such as incidence rates, information about its spread, and the efficacy of measures or regulatory decisions. The COVID-19 pandemic hit Germany at the beginning of 2020. General information in Germany has been delivered on Twitter, with authorities, and particularly other experts such as scientists, science communicators, politicians, and journalists, distributing COVID-19–related tweets [2] and content on the German Twitter network. Both authorities and COVID-19 experts use Twitter to directly share their own insights and opinions with the Twitter community in an unfiltered manner independent of traditional media.

### Prior Work

The number of scientific publications regarding COVID-19 is enormous, including several studies that investigated COVID-19 crisis communication on social media platforms such as Twitter. Crisis communication via Twitter was also studied before the COVID-19 pandemic. For example, surveys investigated Twitter data in regard to crisis communication with a focus on health crises [3], natural disasters [4-7], terrorist attacks [8], or nuclear disasters [9]. Twitter communication during the COVID-19 pandemic has been studied with respect to the following topics: conspiracy theories [10], misinformation and fake news [11,12], stigmatization [13,14], public opinions [15-17], sentiments [18-22], sources of information [23,24], and social networks [25,26]. Likewise, country comparisons [27,28] have been performed. Although previous studies showed that the power of social media, especially Twitter, in crisis communication is very high [29], the Twitter communication behavior of different stakeholder groups has been relatively less studied.

COVID-19 Twitter crisis communication studies have shown that different stakeholders such as scientists, governmental authorities, and politicians, as well as health care professionals tweeted more during COVID-19 [30]. Other studies observed that science-oriented Twitter users contributed to the spread of scientific publications to a great extent [31]. The background of the tweeter has a great impact on the spread of tweets. Existing differences in the popularity of stakeholder groups during COVID-19 have already been documented. Scientists, especially virologists, are now more popular on Twitter than governmental sources [32]. An Italian study analyzed Twitter mentions as a proxy of trust in scientists and reported a loss of trust in science, which was explained by increasing frustration with the COVID-19–induced situations [33].

In the aftermath of the Japan earthquake in 2011, the crisis communication and leadership of the government were neither clear nor apparent on Twitter [34]. Thus, crisis communication by authorities is seen as fundamental during COVID-19 [35] and authorities publish a disproportionally large number of scientifically correct tweets [36]. Previous studies have also shown that during COVID-19, tweets published by authorities are rarely among the most successful tweets [37,38]. This summary of previous studies on the role of authorities and experts indicates that while some findings about the role of different stakeholders exist, direct comparison of communication between authorities and experts against the background of COVID-19 has not yet been addressed.

### Approach

Given this context, the aim of this study was to describe and analyze COVID-19–related tweets by authorities and experts in Germany. This seems to be a necessary task, as COVID-19 is the first health crisis digitally explained and discussed directly by experts, and as such, it competes with the official crisis communication of authorities for attention and coverage. Tweet spread, as our variable of interest, was measured by retweet and like counts. To compare the content of stakeholder tweets and to explain the spread of tweets, the intrinsic message features of “structure,” “content,” and “style” of COVID-19 tweets were compared. Variables related to structure are those capturing whether tweets consist of hashtags, images, URLs, and mentions. With regard to the content features of tweets, Vos et al [3] distinguished four different content categories based on prior work on Twitter risk communication against the background of an infectious disease, hazard content, and fear appeals: severity, susceptibility, efficacy, and technical information. This study builds on this work and further expands the content variables with social, politics, and other categories to best capture COVID-19–specific content and to study similarities and differences in crisis communication. Variables related to style are those using first- or second-person words. Negative binomial regressions were performed to evaluate the spread of tweets, focusing mainly on the number of retweets but also on the number of likes of COVID-19 tweets, and to describe the differences in spread in crisis communication based on 39 German authorities and experts.

## Methods

### Tweet Collection and Data Cleaning

The data set consisted of tweets from 39 German public authorities and experts. The selection of the 39 German authorities and experts was informed by the importance of authorities and experts during the COVID-19 crisis and their visibility in the German discussion. Additionally, these public authorities and experts are active users on Twitter. These stakeholders included 21 authorities and 18 experts. The Federal Ministry of Health and The Robert Koch Institute (RKI) in Berlin were included among the authorities. The expert group consisted of virologists, science communicators, physicians, and other scientists. Multimedia Appendix 1 shows a list of all 39 accounts included in this study.

Tweets of the 39 Twitter accounts were retrieved with a Twitter application programming interface (API) account of the authors. Using tokens, timelines of all stakeholders were retrieved on January 15, 2021. Data analysis was carried out using RStudio (version 1.31093 for Windows) and additional code packages as well as Excel for Microsoft 365 MSO. When creating a Twitter account, users confirm that their tweets are public and can be analyzed by third parties [39]. Using Twitter data for crisis communication analysis is a standard procedure and has been carried out in the context of previous crisis situations, as indicated in the Introduction.

**Figure 1:**
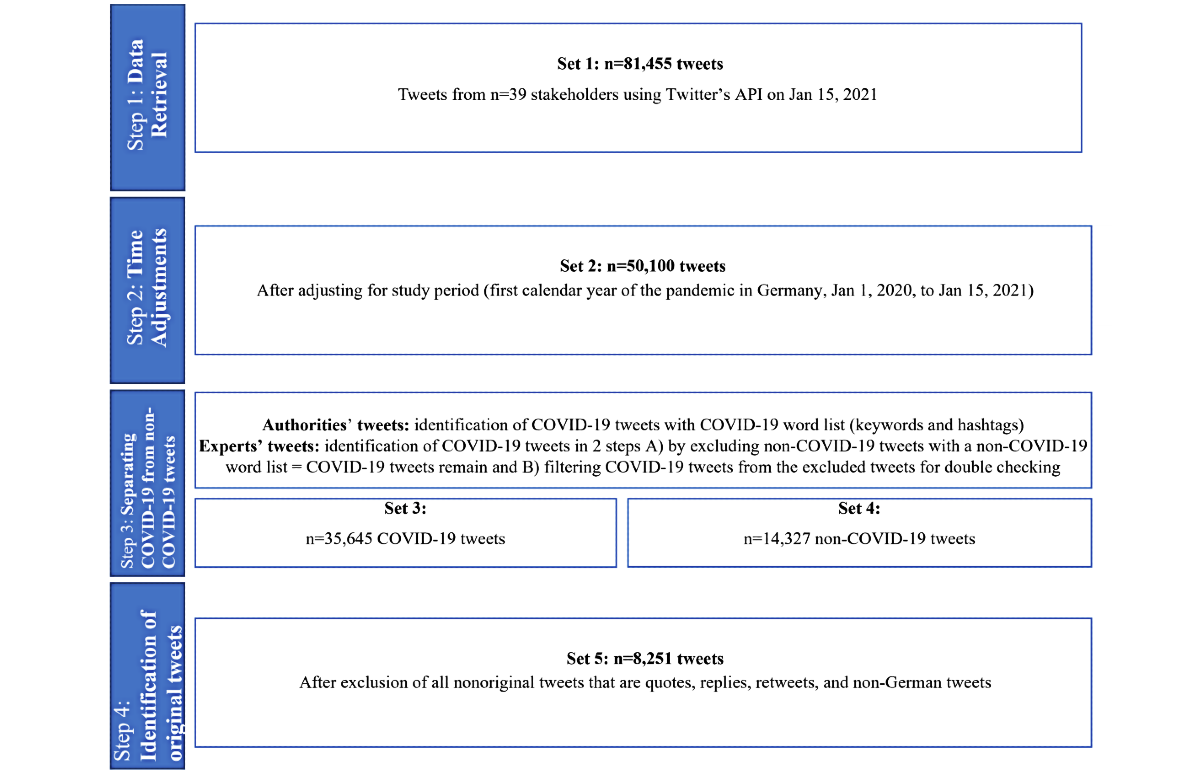
PRISMA (Preferred Reporting Items for Systematic Reviews and Meta-Analyses) diagram of Twitter data adjustment steps. API: application programming interface.

Data retrieval led to 81,455 tweets from authorities and experts. This first data set of 81,455 tweets needed further adjustments. Figure 1 shows the process of data adjustments, including the first step of data retrieval over the Twitter API.

The time frame of tweet analysis was set from January 1, 2020, to January 15, 2021, to capture the first calendar year of the pandemic. However, as vaccinations had just started in Germany at the end of December 2020, and discussions about vaccinations were at a high, the time frame was expanded to January 15. Therefore, as a second step, the data set was adjusted by time so that only tweets from January 1, 2020, to January 15, 2021, were included for the analysis of COVID-19 crisis communication within the first year of the pandemic, leaving 50,100 tweets.

Next, it was necessary to filter COVID-19–related tweets and exclude tweets with other content. To achieve this, COVID-19–related tweets were identified with filter words based on COVID-19 keywords and hashtags. At first glance, tweets of authorities and experts appeared to be very different, and therefore a different filter strategy was used for the two groups. As authorities’ tweets were characterized by the use of hashtags and keywords, COVID-19 tweets were filtered in one step. A COVID-19 word and hashtag list was compiled with 1282 words and hashtags. This list contains a wide spectrum of COVID-19–related words, including different spellings (eg, “corona case,” “corona cases”).

Comparatively, few of the experts’ tweets used hashtags and keywords. As the group of experts’ main expertise is COVID-19, it was assumed that most of these tweets were related to the pandemic; therefore, COVID-19 tweets were identified in two filter steps. In the first filter step, non-COVID-19 tweets were filtered with a non-COVID-19 list of words and hashtags, containing 5789 non-COVID-19–related words (eg, “Navalny,” “Neanderthals”). In this way, most of the COVID-19 experts’ tweets remained in the sample. In the second filter step, the excluded experts’ tweets were filtered again using the COVID-19 filter to capture the experts’ tweets dealing with non-COVID-19 and COVID-19–related topics in one tweet (eg, cancer and COVID-19). Overall, 35,645 COVID-19–related tweets were obtained, 14,624 of which were published by authorities and 21,021 of which came from experts. Authorities tweeted more about non-COVID-19 topics with 13,100 tweets compared to 1157 non-COVID-19 tweets from experts. The complete COVID-19 and non-COVID-19 word lists to separate the tweets are available in Multimedia Appendix 2.

For the content analysis, this study focused on original tweets only. These were tweets written and posted directly from the stakeholders, which excluded replies, retweets, and quotes, as well as tweets in the English language. Thus, COVID-19 tweets were subsequently reduced by quotes, retweets, and replies, leaving 8251 original COVID-19 tweets for content analysis. Of these original COVID-19 tweets, 5432 were from authorities and 2819 were from experts. This last filter step of reducing the data to original tweets illustrated that experts were extremely involved in the COVID-19 discussion on Twitter, with a high share of replies, quotes, and retweets in the broader data set.

### Descriptive Analysis

Based on the text corpus of all COVID-19 tweets by authorities and experts published within the time frame, we first analyzed descriptive statistics of the tweets (eg, their retweets and likes). Content analysis was then performed. German tweets of authorities and experts were used in text form. For regressions explaining the spread of tweets, retweet and like counts were used as dependent variables.

Some of the explanatory model variables to explain the number of retweets and likes were generated following a previous study [3]. Variables related to structure were those capturing whether tweets feature hashtags, images, URLs, and mentions (see [1]). Four dummy variables indicate whether a tweet contained either a hashtag, an image, a URL, or a mention, respectively.

The four content categories of severity, efficacy, susceptibility, and technical information from a previous study [3] were adjusted and extended to seven categories so as to capture the specific aspects of COVID-19 in this study. The dummy variable severity indicates tweets containing information about the severity of the COVID-19 illness, its seriousness, and symptoms, as well as the spread of the virus without a specific location. The dummy variable susceptibility indicates whether a tweet features region-specific information (eg, about incidences and about high-risk subpopulations). Efficacy refers to tweets that give information to help people cope with the disease, for example by applying preventative measures such as washing hands, social distancing, and vaccination. The variable technical information indicates tweets containing biological-technical information related to the technical spreading mechanism of the virus and/or referring to research organizations, research(ers), and COVID-19 studies. The next dummy variable was social, which was used for tweets containing information about the social consequences of the pandemic, such as home schooling or COVID-19 deniers. Politics was the sixth content variable that captured tweets containing information about political consequences such as short-term allowance or regulations. The last category, “other,” was for all other COVID-19–related tweets that could not be attributed to one of the six previous categories.

Tweets were assigned to specific content categories by comparing single words in the tweets with word lists reflecting specific content. Whether or not a tweet contained information about severity was established by comparing tweet words with a list of 521 specific severity words and hashtags. The list to detect susceptibility tweets contained 99 typical words and hashtags in the context of COVID-19 susceptibility. The efficacy word and hashtag list included 451 typical words. Technical information tweets were generated based on a list of 173 COVID-19 technical information words and hashtags. The word list for politics had 124 words and hashtags. Due to the nature of the word lists, tweets could simultaneously be categorized into different categories. The word lists for all categories are provided in Multimedia Appendix 2. Prior to categorization, tweets were adjusted by removing German stop words that did not contain relevant information themselves (eg, “or”). Two coders independently defined the word lists for the seven categories based on the positive COVID-19 filter. After initial coding of 70.50% of the words and consultation with the project initiator, the coding was completed. Coding of the tweets into categories was performed with an R markdown written specifically for the seven categories. Based on the occurrence of words, seven content dummy variables were created.

For the style intrinsic tweet feature, we again followed Vos et al [3] considering whether the tweet was written in the first or second person. Accordingly, dummy variables were created whenever a tweet was written in the first or second person, respectively. Vos et al [3] also considered whether the tweet used a retweet request. The COVID-19 tweets under study only rarely used this style element; thus, this element was neglected.

As it can be expected that retweets and likes would be higher for users with many followers, the followers count was included as another explanatory variable.

### Negative Binomial Regression Analysis

Negative binomial regressions were used to explain the spread of a tweet. Negative binomial regressions are suitable when the dependent variable is a count variable with many zero observations. Tweet spread (ie, the dependent variable) was measured by either the retweet or like count. These counts were retrieved together with the tweets. In the context of this study, a retweet meant that a follower of the 39 stakeholders republished the stakeholder’s tweet. The tweet content was therefore distributed by all users who retweeted that tweet. The more the retweets, the more the original tweet was spread. The like count was the second indicator used to measure the spread of tweets. Likes are an indicator of popularity. It is argued that retweets reflect more engagement as they indicate more interaction compared to likes. A retweet means that the user is sharing the stakeholder’s content with the possibility to comment. This is not the case for likes. Overall, four different regressions were executed: four negative binomial regressions to explain the number of retweets (and likes), separated by authorities and experts. For the retweet and like counts, the dependent variables did not follow a normal distribution and the variance exceeded the mean. The data can be regarded as overdispersed, and therefore the negative binomial regression was the best choice to explain the count variables.

## Results

### Descriptive Analysis

**Figure 2:**
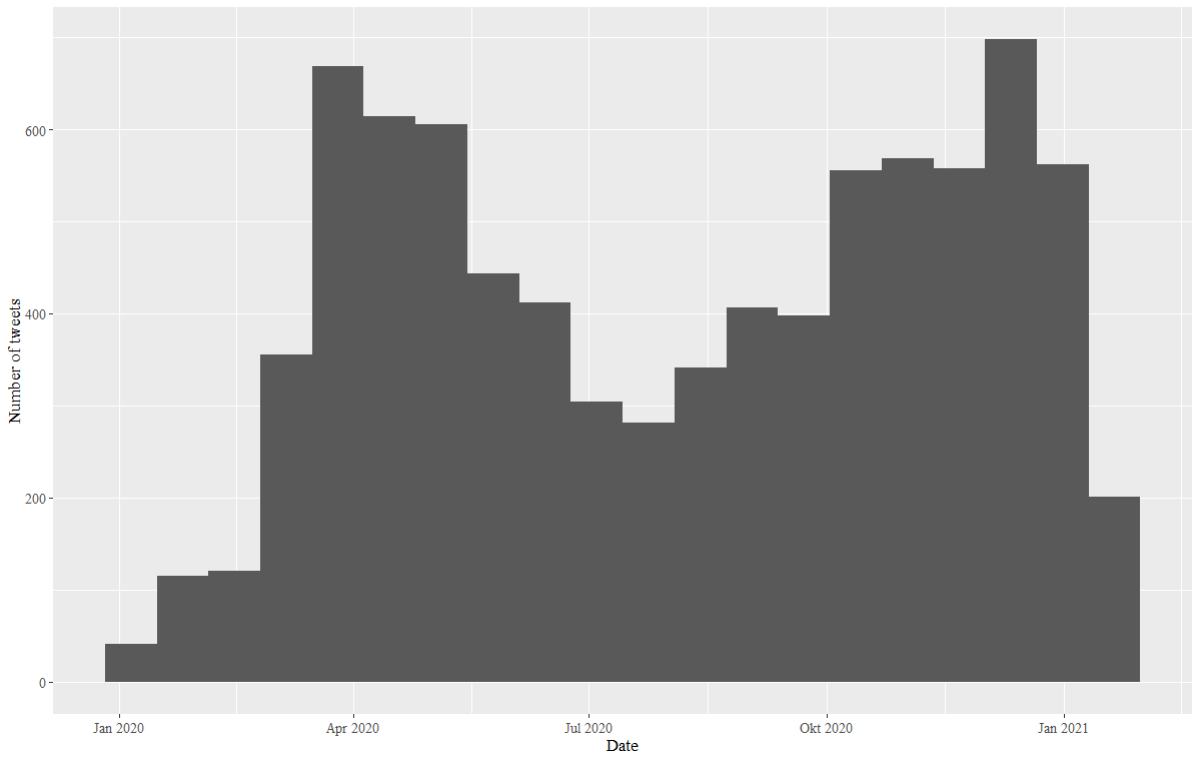
Number of original COVID-19 tweets over time (January 1, 2020, to January 15, 2021) in Germany (N=8251).

A list of the Twitter stakeholders analyzed and descriptive statistics for their numbers of followings and followers are given in Multimedia Appendix 1. The development of the COVID-19 crisis communication of the 39 experts and authorities is shown in Figure 2.

Strikingly, the number of COVID-19 tweets from January 1, 2020, to January 15, 2021, paralleled the development of COVID-19 cases in Germany, including the first two waves (March/April and beginning of October) during the sample period. The time of lower COVID-19 cases was also mirrored in the number of tweets, with a smaller number of tweets in the summer of 2020. This effect, namely the parallelism of tweet activity and (COVID-19) incidences, has already been documented elsewhere [28,40]. Moreover, both authorities and experts increasingly tweeted about COVID-19 when considering the number of tweets.

Table 1 shows the descriptive statistics for the dependent and independent variables in the regression models.

The descriptive statistics in Table 1 revealed strong differences in COVID-19 crisis communication between authorities and experts in Germany during the first year of the pandemic. We further focus on the differences in numbers of followings, followers, retweets, and likes as proxies of the distribution of COVID-19 communication.

On average, experts had 814 followings, representing 1.21 times the number of followings compared with that of authorities, with an average of 671. Although these numbers show that experts had a bigger network, the mean of followings did not differ significantly between the two groups (t_37_=0.54, *P*=.60). The number of followings shows that stakeholders were willing to communicate with others. Among the authorities, the Helmholtz Association of German Research Centres (@HelmholtzG) had the highest number of followings (see Multimedia Appendix 1). In the group of experts, expert E9 had the highest number of followings, with more than 3200 followings. The regional office of the World Health Organization in Europe (@WHO_DE) had the lowest number of followings in the group of authorities and expert E11 had the lowest number in the expert group with only 2 followings.

The number of followers exceeded the number of followings to a great extent. Among the authorities, RKI had the highest number of followers, with more than 430,000. In the group of experts as well as overall, expert E8 had the biggest follower network, with more than 650,000 followers (as of January 15, 2021). Less than 1000 followers had the Max Planck Institute for Infection Biology in the group of authorities. In the experts’ group, expert E1 had the lowest number of followers (1270). On average, experts had 99,059 followers, representing 1.31 times the number of followers for authorities with an average of 75,817 followers. However, this differences in mean followers was also insignificant (t_37_=0.52, *P*=.60).

Experts were considerably more frequently retweeted with 7-fold (7.03) more retweets than authorities. On average, a tweet by authorities was retweeted 30.7 times, whereas tweets originating from COVID-19 experts were retweeted 215.7 times. This difference in mean retweets by stakeholder group was statistically significant (*t*_8,249_=26.94, *P*<.001). The dominance of experts became even more obvious when comparing mean likes: authorities’ tweets were liked 90.8 times and the experts’ tweets reached an average of 1257 likes, representing a 13.9 times increase for the experts’ tweets. The difference in mean likes between authorities and experts was highly significant (*t*_8,249_=31.27, *P*<.001).

**Table 1:** Descriptive statistics for variables used in the *t* tests and regression models (N=8251 tweets).

Variable	Description	Authorities (n=5432), mean (minimum, maximum)	Experts (n=2819), mean (minimum, maximum)
Retweet count	Metric variable for the number of retweets (dependent variable in negative binomial regression)	30.70 (0, 9982)	215.70 (0, 8457)
Like count	Metric variable for number of likes (dependent variable in negative binomial regression)	90.80 (0, 44,274)	1257 (0, 63,002)
Hashtag	Structural dummy variable that is 1 if a tweet has a hashtag	0.92 (0, 1)	0.19 (0, 1)
Images	Structural dummy variable that is 1 if a tweet uses images	0.69 (0, 1)	0.17 (0, 1)
URL	Structural dummy variable that is 1 if a tweet uses a URL	0.71 (0, 1)	0.71 (0, 1)
Mention	Structural dummy variable that is 1 if a tweet carries a mention	0.40 (0, 1)	0.24 (0, 1)
Severity	Content dummy variable that is 1 if a tweet contains words describing COVID-19 severity	0.75 (0, 1)	0.52 (0, 1)
Susceptibility	Content dummy variable that is 1 if a tweet contains words describing susceptibility to COVID-19	0.22 (0, 1)	0.05 (0, 1)
Efficacy	Content dummy variable that is 1 if a tweet contains words describing efficacy measures	0.35 (0, 1)	0.28 (0, 1)
Technical information	Content dummy variable that is 1 if a tweet contains a word related to technical virus information	0.04 (0, 1)	0.07 (0, 1)
Social	Content dummy variable that is 1 if a tweet contains words describing the social consequences of COVID-19	0.13 (0, 1)	0.08 (0, 1)
Politics	Content dummy variable that is 1 if a tweet contains words describing the political consequences of COVID-19	0.11 (0, 1)	0.04 (0, 1)
Other	Content dummy variable that is 1 if a tweet contains a word that cannot be attributed to other content variables	0.03 (0, 1)	0.31 (0, 1)
First person	Style dummy variable that is 1 if a tweet uses first-person words	0.27 (0, 1)	0.44 (0, 1)
Second person	Style dummy variable that is 1 if a tweet uses second-person words	0.04 (0, 1)	0.06 (0, 1)
Followers count	Metric variable as the number of followers per Twitter user	75,817 (971, 435,392)	99,059 (1270, 657,292)
Followings count	Metric variable as the number of followings per Twitter user	671 (38, 3424)	813.90 (2, 3293)

Considering the top users in crisis communication, previous studies evaluated the concentration of retweets among single users. For COVID-19 in Germany, two experts and the Federal Ministry of Health were responsible for 70.26% (544,418/774,865) of all retweets, and as such were the COVID-19 influencers in Germany on Twitter.

As seen in Table 1, the composition of COVID-19 tweets (N=8251) differed largely between authorities (n=5432 tweets) and experts (n=2819 tweets). Authorities strongly used structural content elements such as hashtags, images, URLs, and mentions, and clearly followed the common rules of general successful social media communication. It can be suggested that this is due to fact that authorities’ tweets are published by their own social media departments who follow the rules of social media designs. Out of the 5432 tweets by authorities, 91.64% (n=4978) and 69.04% (n=3750) used hashtags or images, respectively, whereas only 19.44% (n=548) and 16.89% (n=476) of the 2819 experts’ tweets used these elements. The proportion of tweets containing URLs was equal for authorities and experts. Mentions were included in 39.62% (n=2152) of the tweets by authorities’ and in 24.26% (n=684) of the tweets by experts. Overall, the experts’ use of structural elements was much lower than that of authorities. Experts clearly published their tweets on their personal accounts and did not spend time structuring the tweets in the same way as the official social media divisions of authorities.

There were also differences between the stakeholders under study with regard to the content of the tweets, with the strongest difference observed for severity. Overall, 75.00% (n=4074) of authorities’ tweets referred to the severity of COVID-19, such as with reference to symptoms, whereas only 51.47% (n=1451) of experts’ tweets were categorized into severity. In 22.22% (n=1207) of the authorities’ tweets, there were words referring to susceptibility and regional information related to COVID-19, whereas this was the case in only 4.68% (n=132) of the experts’ tweets. With regard to efficacy information, the tweets of experts and authorities were similar with 34.70% (n=1885) and 27.49% (n=775) of tweets including this content, respectively. Tweets bearing technical information of the spread of the virus and references to scientific findings were more frequent for experts (n=188, 6.66%) than for authorities (n=224, 4.12%).

More of the authorities’ tweets referred to the social consequences (n=719, 13.24%) and political consequences (n=617, 11.36%) of COVID-19 than the experts’ tweets (n=225, 7.98% and n=104, 3.68%, respectively). The content category “other,” which refers to words in the tweets that cannot be categorized by any of the category word lists, explains the differences observed: 31.80% (n=896) of experts’ tweets and only 3.73% (n=203) of authorities’ tweets were related to content that was not captured by the previous categories. Thus, the tweets of the experts were much simpler and did not use that many clearly detectable keywords, favoring more colloquialisms than authorities. An example is the tweet of a German expert “Sehr gut” (“very good”) with additional links to external information. Categorization of these tweets by quantitative text analysis was impossible. Taken together, the findings from analysis of the structural variables show that authorities were much better in using structure, hashtags, and keywords.

The tweets of experts and authorities also differed with regard to the style variables: 26.82% (n=1457) of authorities’ tweets and 43.70% (n=1332) of experts’ tweets used first-person words, whereas 3.73% (203) of authorities’ tweets and 5.57% (n=157) of experts’ tweets used second-person words. The fact that experts used the second person slightly more often than authorities indicates that they interacted more with other Twitter users.

### Negative Binomial Regression Analysis

Table 2 shows the results of the negative binomial regressions for authorities and experts on the retweet count of COVID-19 tweets.

The results for the like count regressions were similar, which are shown in Multimedia Appendix 3.

Table 2 provides the estimation results (incidence rate ratios [IRRs], *Z* values, and *P* values) separately for experts and authorities. IRRs were calculated from the estimated parameters of the negative binomial regression. They are easier to interpret than estimated values for negative binomial regressions. The IRR compares the impact of a (dummy) variable relative to the reference category, given that all other model variables are held constant. When the *Z* value from the negative binomial regression is positive, the direction of the effect is positive, whereas when the *Z* value is negative, the direction is negative.

**Table 2:** Negative binomial regression to explain the retweet count of COVID-19 tweets for authorities and experts (N=8251 tweets).

Variables		Authorities^a^			Experts^b^		
		IRR^c^	*Z*	*P* value	IRR	*Z*	*P* value
Model variable: constant		16.69	30.57	<.001	71.95	61.37	<.001
**Structural variables**							
	Hashtag	0.64	–6.92	<.001	1.11	1.56	.12
	Images	1.06	1.32	.19	1.06	0.87	.38
	URL	0.82	–4.81	<.001	0.76	–4.27	<.001
	Mentions	0.81	–5.45	<.001	0.73	–5.27	<.001
**Content variables**							
	Severity	1.40	8.09	<.001	1.18	3.34	<.001
	Susceptibility	1.02	0.46	.65	1.15	1.21	.23
	Efficacy	1.34	8.63	<.001	1.10	1.71	.09
	Technical information	1.45	4.23	<.001	1.00	0.06	.95
	Social	1.24	4.05	<.001	1.27	2.69	.01
	Political	0.71	–6.12	<.001	0.87	–1.12	.26
**Style variables**							
	First person	0.93	–1.80	.07	1.10	0.02	.99
	Second person	1.88	6.96	<.001	1.03	0.23	.82
Other: followers count		1.00	28.74	<.001	1.00	25.99	<.001

^a^Authorities: –2 log-likelihood=–44365.18; Akaike information criterion=44,395; null model logistic regression *χ^2^*=1854.8 (*P*<.001); McFadden pseudo *R²=*0.04.

^b^Experts: –2 log-likelihood=–33,752.49; Akaike information criterion=33,782; null model logistic regression *χ^2^*=956,66 (*P*<.001); McFadden pseudo *R²*=0.03.

^c^IRR: incidence rate ratio.

The regression constant for both authorities and experts was significant and positive. The first set of explanatory variables included four dummy variables that recorded whether a tweet used a hashtag, image, URL, or a mention, and as such captured structural elements of intrinsic message features. The number of retweets for authorities’ COVID-19 tweets that used a hashtag was 0.64 the number of retweets for tweets that did not carry a hashtag, indicating a negative impact of hashtags. This result was highly significant (*P*<.001). For experts, the number of retweets was not significantly affected by hashtags (*P*=.12).

When both authorities’ and experts’ tweets had images, the retweet count was not significantly different compared with tweets without an image. Using URLs in an authority’s tweet reduced the success of the tweet with respect to retweet counts: the number of retweets was approximately 0.82 that for authorities compared to tweets without URLs (*P*<.001). Likewise, experts’ tweets using URLs led to a lower number of retweets, with an approximately 0.76 reduction compared to experts’ tweets without URLs (*P*<.001). The usage of mentions reduced the success of retweets for authorities and experts alike. The number of retweets of authorities’ tweets was approximately 0.81 that of authorities’ tweets without mentions (*P*<.001). For experts, the number of retweets was also lower when a mention was present in the tweet, by a factor of 0.73, compared to experts’ COVID-19 tweets without mentions (*P*<.001).

As a second category of intrinsic message features, the effect of different content and themes of tweets was analyzed, considering six content variables: tweets containing words referring to the severity of COVID-19, susceptibility, efficacy, tweets containing technical information about the spread of the virus, as well as tweets containing words referring to social and political consequences of the COVID-19 pandemic. As for the structural categories, there were fundamental differences in the success of COVID-19 crisis communication between authorities and experts. The first content category considered was severity. Authorities’ tweets containing words referring to the severity of COVID-19 were associated with 1.40 more retweets than those of authorities’ tweets that did not refer to severity (*P*<.001). Similarly, for experts, there was a positive and significant effect of severity content on the retweet count, which was approximately 1.18 times that of experts’ tweets not referring to severity (*P*<.001).

The retweet count of authorities’ COVID-19 tweets that contained informative words about susceptibility (eg, regarding SARS-Cov2 at-risk groups) was not significantly different from that of tweets without this information. For experts, there also was no significant impact of susceptibility content on the retweet count. Tweets with efficacy information increased the tweet success for authorities: the retweet count of authorities’ tweets with efficacy content was approximately 1.34 times that of tweets without this content (holding all other model variables constant). This effect was highly significant (*P*<.001). For experts’ tweets with efficacy content, there was no significant difference in the retweet count compared to that of tweets with no efficacy content.

For authorities, tweets with technical information about the spread of the virus and other scientific findings were positively associated with retweet count. For authorities, the retweet count was approximately 1.45 that of tweets without this information (*P*<.001). For experts, the retweet count was not significantly different for this content category (*P*=.95).

Tweets that contained words referring to social consequences (eg, lockdown) led to a retweet count for authorities that was approximately 1.24 times (*P*<.001) that of tweets without this information. When experts tweeted about social consequences, the retweet count was approximately 1.27 times (*P*=.01) that of experts’ tweets that did not refer to social consequences. If an authority’s tweet contained references to the political consequences of the pandemic, the retweet count was 0.71 times (*P*<.001) that of tweets without political content. However, there was no significant impact of political words in experts’ tweets on the retweet count (*P*=.26).

The third category of explanatory variables considered the impact of style elements of COVID-19 tweets on the retweet count. COVID-19 tweets written in first-person language had no impact on the retweet count for experts or authorities. However, when authorities’ tweets used second-person language, the retweet count was approximately 1.88 times that of tweets that did not use it (*P*<.001). When experts used second-person words, their retweet count was not significantly different compared to that of tweets not using these words (*P*=.82).

The followers count, as an independent variable, had a highly significant impact on the spread of tweets for both authorities and experts (*P*<.001). Thus, spread of tweets is higher for stakeholders with a larger network.

These results reveal that there are differences and similarities in the determinants of the retweet success of COVID-19 tweets between authorities and experts.

## Discussion

### Main Findings

Overall, this study indicated strong differences between authorities and experts as to what increases the retweet rate of crisis communication regarding COVID-19 on Twitter.

Over the timeframe studied, authorities and experts tweeted increasingly about COVID-19 when considering the number of tweets (see Figure 2). However, authorities tweeted much more frequently about other non-COVID-19–related topics after filtering all tweets of the 39 stakeholders under study for COVID-19 and non-COVID-19 tweets (step 3 in Figure 1). The tweets of experts, with specific knowledge, were much more focused on COVID-19 during the first year of the pandemic in Germany. This focus on COVID-19 might be the reason why experts were perceived as more credible information sources (eg, in terms of numbers of followers) than authorities and received much higher spread on Twitter. Results regarding the types of tweets indicate that experts are much more involved in exchanging information with other Twitter users, as a great number of tweets are replies, retweets, and quotes.

The results of the negative binomial regression validated the descriptive analysis in that fundamental differences in crisis communication were observed between authorities and experts in Germany. These can be traced back to intrinsic message features such as the structural, content, and style features of tweets [3]. Structural elements in COVID-19 tweets, similar to URLs and mentions, were negatively correlated with the number of retweets for both authorities and experts. In addition, authorities’ tweets with hashtags were less retweeted compared to tweets without hashtags. COVID-19 tweets of authorities clearly follow the common rules of successful social media communication. With a higher share of structural elements, crisis communication does need different social media communication standards to make authorities’ crisis communication more successful. Crisis communication must be more immediate, direct, and fast to reach the public and should not be hidden behind those elements. The effect of a direct mention of other Twitter users indicates that the general community of Twitter users was excluded from that specific communication.

Referring to the content of COVID-19 tweets, for authorities, the content categories were mostly positively associated with the retweet count. Content covering susceptibility did not significantly affect spread, whereas information about severity, efficacy, technical information, and tweets about social consequences led to a higher retweet count for authorities.

Content about political consequences of the pandemic led to a lower number of retweets compared to tweets without political information, again for authorities only. This result seems remarkable, especially against the background of crisis communication, because political information is the core element of authorities’ communication. Moreover, tweets referring to technical information about the spread of the virus and research results (eg, regarding vaccine development) led to a higher retweet count for authorities than tweets without these references.

Looking at the COVID-19 tweets of experts, it must be noted that fewer content variables were significant in explaining the retweet count. Only two content variables were significantly and positively correlated with the number of retweets. These were tweets dealing with the severity and social consequences of COVID-19. It is within these categories that the experts’ knowledge was valued by Twitter users.

In contrast to the results regarding the structure of COVID-19 tweets, authorities’ and experts’ tweets using severity information were retweeted more often. For both groups, content about susceptibility was not associated with the retweet count. Information about efficacy and technical information increased the retweet count only for authorities’ tweets. Tweets with content about social consequences led to a higher retweet count for both groups of stakeholders. Strikingly, political content in authorities’ tweets was associated with a lower spread of corresponding tweets.

Style elements in COVID-19 tweets considered first- and second-person–specific words. Authorities should consider increasing tweets written in the second person to directly address users, as the relationship with the retweet count was positive. Style variables were not related to significant differences in retweets of experts. Overall, looking at intrinsic message features of COVID-19 tweets reveals that authorities’ tweets appear to be more designed with the strong use of structural elements such as hashtags and URLs compared with the COVID-19 tweet of experts.

Further, the larger the network of the stakeholders, the larger the retweet count. This indicates that stakeholders need to make great efforts in expanding and maintaining a large network to disseminate their crisis communication messages.

Comparing the results of this study with previous studies on crisis communication prior to the pandemic such as that of Vos et al [3], this study also shows that COVID-19 retweets depend on structural, content, and style variables; the account sending the message; as well as the network of the account (with a significant positive impact of the number of followers on retweet count). Regarding the content of the tweets, Vos et al [3] indicated that tweets dealing with severity of the Zika virus increase how often messages are shared. The same holds true for COVID-19, as this study shows that for both groups of stakeholders, tweets that deal with severity of COVID-19 are retweeted more often compared to tweets not referring to severity. As indicated by Vos et al [3], the Zika virus led to a situation of high ambiguity where little was known about the virus and the information need of the public was high. Accordingly, recommendation by authorities changed as more became known about the virus. The same holds true for COVID-19, especially during the first year of the pandemic.

There are also similarities to the swine flu crisis in 2009, which was studied by Kostkova et al [41], who showed that the number of swine flu cases was linked to the corresponding Twitter activity. We found the same relationship for COVID-19 cases and Twitter activity. Thus, Twitter communication can act as a rapid alert system, and it is possible to observe risk perceptions of the public by analyzing tweets.

Overall, confirming previous studies, there was a strong concentration of retweets in COVID-19 crisis communication. Two experts and the Federal Ministry of Health were responsible for 70.26% (544,418/774,865) of all retweets, and as such were COVID-19 influencers in Germany on Twitter. A study on the nuclear crisis of Fukushima [9] showed that 80.30% of retweets originated from only 2.00% of users.

This study also confirms previous Twitter-specific crisis communication studies in showing that all stakeholders tweeted more about COVID-19 over the course of time [30]. Caro [32] stated that virologists are very famous on Twitter. For Germany, we can confirm this finding as there is one virologist with by far the highest number of followers who belonged to the group of COVID-19 influencers identified in this study. The result that authorities are not very popular and that experts are the preferred information source over authorities has been documented elsewhere [32,37]. This study confirms this pattern for COVID-19 based on the significantly higher number of followers for experts indicating higher popularity. Rao et al [38] showed that alarming tweets of US health authorities were retweeted less often compared to reassuring tweets. Although this study did not compare alarming vs reassuring tweets, we found that tweets of authorities and experts dealing with the severity of COVID-19 are retweeted more often, whereas there was no significant effect of susceptibility tweets on retweets in Germany.

### Implications

Overall, there are several differences in crisis communication between authorities and experts regarding COVID-19 in Germany: experts have a larger network of followings and followers, receive a much higher spread via retweets and likes, and engage to a larger extent more directly with Twitter users about COVID-19 themes compared to authorities (which became more obvious after filtering out quotes, replies, retweets, and non-German tweets). Regarding intrinsic message features, the fact that experts use fewer structural and style elements in tweets than authorities and exceed them by far in spread indicates that other aspects such as sympathy, reputation, publicity, reliability, general media presence, and directness/speed in communication are more important for crisis communication on Twitter.

Both groups should tweet more about specific COVID-19–related topics. Tweets with content about the severity of COVID-19 had more retweets compared with tweets that did not make severity references. However, it seems advisable to prevent alarmism in the public. More research is needed to determine how the results can be translated one by one to authorities’ crisis communication, such as when it comes to directness in crisis communication versus preventing alarmism.

### Limitations

It must be noted that Twitter users are not representative of the overall German population. By contrast, only few, albeit more educated, people use Twitter in Germany [42]. Considering the two groups under study, authorities and experts, there were a few stakeholders who hardly contributed to COVID-19 crisis communication during the first year of the pandemic. Moreover, some interesting crisis communication stakeholders on Twitter in Germany emerged only after the study started. The separation between experts and authorities in this study should have been more specific or analyzed with more subgroups. In particular, differentiating authorities as science organizations vs organizations with sovereign duties seems promising for future research.

It is important to note again that the comparison between the groups of experts and authorities is limited. The modes of crisis communication of these two groups are different. For example, authorities have many other communication channels (online and offline) that they can use for crisis communication, such as press conferences. Moreover, it can be questioned to what extent authorities use Twitter to inform the public in general or more specific target groups such as journalists or experts themselves. This study only analyzed tweets written in German. To communicate within the scientific community, experts especially use English; however, these tweets were not analyzed.

The study results further indicate that there might be other determinants for the success of specific stakeholders on Twitter, which cannot be observed by only using Twitter data, such as sympathy, reputation, publicity, reliability, and a general media presence during the study period. Overall, the tweets were downloaded on a specific date and as such represent a snapshot of events. The data, retrieved on January 15, 2021, cover a singular period amidst an ongoing crisis with an unforeseeable ending and are also designed to be analyzed retrospectively.

### Conclusion

Twitter data represent a powerful information source and are suitable for crisis communication in Germany regarding COVID-19. Some important results can be highlighted. COVID-19 tweet activity mirrors the COVID-19 case numbers in Germany. Both authorities’ and experts’ COVID-19 tweets have higher spread when they are plain and for authorities when they address the public directly. Experts’ success in crisis communication on Twitter outweighs the spread of authorities by far. Experts are more valued as an information source in the pandemic situation than authorities. For authorities, it appears difficult to win recognition during a crisis when their crisis communication is not only related to the specific crisis. Authorities should consider developing separate accounts on Twitter and using these accounts for more targeted communication.

## Appendix

Multimedia Appendix 1 List of stakeholders and number of followings and followers.

Multimedia Appendix 2 Complete word lists.

Multimedia Appendix 3 Additional binomial regression results on likes count.

## Abbreviations

API: application programming interface
IRR: incidence rate ratio
RKI: Robert Koch Institute
