# Supplementary material for: The Spread of COVID-19 Crisis Communication by German Public Authorities and Experts on Twitter: Quantitative Content Analysis
Source: JMIR Public Health Surveill. 2021 Dec 22;7(12):e31834. doi: 10.2196/31834 (PMC8698804; doi:10.2196/31834)
Supplement: Multimedia Appendix 1 [file publichealth_v7i12e31834_app1.docx]

Multimedia Appendix 1: [List of stakeholders and number of followings and followers]

This table lists Twitter stakeholders and the number of followings and followers as of January 15, 2021.

|  |  | **Followings (former Friends)** | **Followers** |
| --- | --- | --- | --- |
| **Authorities** |  |  |  |
|  | BBK (Federal Office of Civil Protection and Disaster Assistance) @BBK_Bund | 465 | 45,428 |
|  | BfR (The German Federal Institute for Risk Assessment) @BfRde | 387 | 10,695 |
|  | BMG (Federal Ministry of Health) @BMG_Bund | 994 | 251,363 |
|  | BW (The State Government of Baden-Württemberg) @RegierungBW | 1,101 | 77,931 |
|  | BZgA (Federal Center for Health Education) @bzga_de | 469 | 70,317 |
|  | Charite (The Charité Berlin – Charité University Medical Department Berlin) @ChariteBerlin | 229 | 47,170 |
|  | HH (Hamburg Senate) @Senat_Hamburg | 177 | 24,515 |
|  | HelmholtzG (Helmholtz Association of German Research Centres) @helmholtz_de | 3,424 | 86,009 |
|  | HZI (Helmholtz Centre for Infection Research) @Helmholtz_HZI | 1,149 | 8,742 |
|  | LAGeSo (Regional Office for Health and Social Affairs, Berlin) @LaGeSo_Berlin | 479 | 2,280 |
|  | Leopoldina (German National Academy of Sciences) @Leopldina | 515 | 14,466 |
|  | LSA (Federal State Government of Saxony-Anhalt) @sachsenanhalt | 247 | 14,219 |
|  | Max Planck (Max Planck Society) @maxplanckpress | 1,678 | 163,786 |
|  | MPIIB (Max Planck Institute for Infection Biology) @mpiib_berlin | 100 | 971 |
|  | MUC (City of Munich) @StadtMuenchen | 97 | 239,442 |
|  | NDS (Federal State of Lower Saxony) @NdsLandesReg | 404 | 11,833 |
|  | PEI (Federal Institute for Vaccines and Biomedicines) @PEI_Germany | 662 | 7,520 |
|  | RKI (The Robert Koch Institute) @rki_de | 123 | 435,392 |
|  | RLP (Federal State Government of Rheinland-Pfalz) @rlpNews | 701 | 73,114 |
|  | WHO_DE (World Health Organization, Regional Office for Europe) @WHO_Europe_de | 38 | 2,813 |
|  | WPK (The German Science Journalists’ Association) @wpk_daily | 652 | 4,145 |
| **Experts** |  |  |  |
|  | E1 | 395 | 1,270 |
|  | E2 | 276 | 1,490 |
|  | E3 | 673 | 2,676 |
|  | E4 | 467 | 28,635 |
|  | E5 | 1,104 | 7,525 |
|  | E6 | 111 | 16,763 |
|  | E7 | 57 | 67,554 |
|  | E8 | 121 | 657,292 |
|  | E9 | 3,293 | 35,310 |
|  | E10 | 2,036 | 36,973 |
|  | E11 | 2 | 76,697 |
|  | E12 | 1,609 | 113,520 |
|  | E13 | 2,191 | 19,261 |
|  | E14 | 261 | 345,888 |
|  | E15 | 982 | 230,353 |
|  | E16 | 102 | 24,679 |
|  | E17 | 405 | 95,254 |
|  | E18 | 566 | 21,923 |
