# Supplementary material for: The Spread of COVID-19 Crisis Communication by German Public Authorities and Experts on Twitter: Quantitative Content Analysis
Source: JMIR Public Health Surveill. 2021 Dec 22;7(12):e31834. doi: 10.2196/31834 (PMC8698804; doi:10.2196/31834)
Supplement: Multimedia Appendix 3 [file publichealth_v7i12e31834_app3.docx]

Multimedia Appendix 3: [Additional binomial regression results on likes count]

Table 3. Negative binomial regression to explain likes count of COVID-19 tweets for authorities and experts (n = 8,251).^a-c^

|  |  | Authorities^a^ | Experts^b^ |
| --- | --- | --- | --- |
|  | Variable names | IRR^c^ / z -value | IRR^c^ / z-values |
| **Model variables** |  |  |  |
|  | Constant | 40.79 / 42.47 (*P*<.001) | 555.47 / 99.25 (*P*<.001) |
| **Structural variables** |  |  |  |
|  | Hashtag | 0.-97 / -0.43 (*P*=.67) | 0.91 / -1.56 (*P*=.12) |
|  | Images | 1.04 / 1.06 (*P*=.29) | 0.87 / -2.20 (*P*=.03) |
|  | URL | 0.58 / -13.98 (*P*<.001) | 0.46 / -13.44 (*P*<.001) |
|  | Mentions | 0.99 / -0.22 (*P*=.82) | 0.74 / -5.64 (*P*<.001) |
| **Content variables** |  |  |  |
|  | Severity | 1.31 / 6.80 (*P*<.001) | 0.93 / -1.58 (*P*=.11) |
|  | Susceptibility | 0.91 / -2.34 (*P*=.02) | 1.01 / 0.11 (*P*=.91) |
|  | Efficacy | 1.26 / 6.51 (*P*<.001) | 1.11 / 2.11 (*P*=.03) |
|  | Spread | 1.21 / 2.34 (*P*=.02) | 0.83 / -2.07 (*P*=.03) |
|  | Social | 1.08 / 1.59 (*P*=.11) | 1.17 / 1.99 (*P*=.04) |
|  | Political | 0.60 / -9.72 (*P*<.001) | 1.04 / 0.36 (*P*=.72) |
| **Style variables** |  |  |  |
|  | First person | 0.91 / -2.33 (*P*=.02) | 1.24 / 4.78 (*P*<.001) |
|  | Second person | 1.97/ 7.88 (*P*<.001) | 1.50 / 4.12 (*P*<.001) |
| **Other explanatory variables** | Followers count | 1.00 / 34.84 (*P*<.001) | 1.00 / 35.74 (*P*<.001) |

^a^Authorities: -2 Log-likelihood = -55509.12, AIC = 55539, null model LR Chi^2^ 2392.7 (p<0.000) (df = 13), McFadden’s Pseudo R²= 0.04

^b^Experts: -2 Log-likelihood = -44225.39, AIC = 44255, null model LR Chi^2^ 1420.6 (p<0.000) (df = 13), McFadden’s Pseudo R²= 0.03

^c^IRR: Incidence rate ratio
